# Thermoregulatory reactions of female buffaloes raised in the sun and in the shade, in the climatic conditions of the rainy season of the Island of Marajó, Pará, Brazil

**DOI:** 10.3389/fvets.2022.998544

**Published:** 2022-09-13

**Authors:** Jamile Andréa Rodrigues da Silva, Messy Hennear de Andrade Pantoja, Welligton Conceição da Silva, Jean Caio Figueiredo de Almeida, Rafaella de Paula Pacheco Noronha, Antônio Vinicius Corrêa Barbosa, José de Brito Lourenço Júnior

**Affiliations:** ^1^Department of Health and Production, Institute of Animal Health and Production, Federal Rural University of the Amazon, Belem, Brazil; ^2^Department of Veterinary Medicine, Institute of Veterinary Medicine, Federal University of Pará, Belem, Brazil; ^3^Department of Cyberspace Institute, Cyberspace Institute, Federal Rural University of the Amazon, Belem, Brazil

**Keywords:** heat, climate, thermoregulation, buffaloes, productivity

## Abstract

Buffaloes are well-adapted to hot, humid climates and muddy terrain, however they show signs of thermal discomfort when exposed to direct sunlight due to their specific structural features such as dark skin, small number of sweat glands/skin area and thick skin. Epidermis layer of the skin, making heat dissipation difficult. The study aimed to evaluate the thermal comfort of female buffaloes raised in traditional and silvopastoral systems, on the island of Marajó, Pará, during the rainy season of the year. The experiment was carried out in Cachoeira do Arari, Retiro Grande, Marajó, Pará (00°55'37.814424”S 48°43'48.143060”W). Twenty female Murrah buffaloes, aged between 2 and 3years and average weight of 282 ± 29 kg, were used. They were fed on pasture and divided into two groups: WS group (with shade) and NS group (without shade). The WS group (*n* = 10) remained grazing in a silvopastoral system, with access to the shade of red Jambeiro (*Syzygium malaccense*) trees. The NS group (*n* = 10) was kept in grazing, in a traditional system, without access to the shade of trees or shades. The physiological variables rectal temperature (RT), respiratory rate (RR) and body surface temperature (BST) (forehead, left side of the thorax and left flank) were measured at 6:00, 10:00, 14:00, 18:00, and 22:00 h. At the same times, data on air temperature, relative air humidity, wind speed (WSP), solar radiation (SR) and Temperature and Humidity Index (THI) were also recorded. THI, WSP and SR were higher at 14:00 h (*P* < 0.05). At 10:00, 14:00, and 18:00 h there was difference of RT between treatments (*P* < 0.05), where animals of the NS group had the highest values. In the NS group, the highest value of RT was observed at 14:00 h (39.38 ± 0.43°C), when THI and SR were higher. At 10:00 and 14:00 h, BST was higher in the NS group (*p* < 0.05), of 34,55 ± 1.03 and 35.35 ± 1.51°C, respectively, and both groups had the highest value of BST at those same times. There was difference of the RR between treatments at 10:00 h and 14:00 h (*p* < 0.05), where the highest values were found in the NS group (33.71 ± 7.58 e 50.40 ± 18.41 mov./min., respectively). Even in the rainy season of the year, the climatic conditions of Ilha do Marajó are unfavorable to the welfare of buffaloes, especially at 2:00 pm, when the SR is higher. Thus, the use of the system with access to shade, such as silvopastoral, is recommended, in order to provide greater thermal comfort to animals and improve their productivity.

## Introduction

The introduction of the buffalo herd in Brazil began in 1890, on the island of Marajó, Pará, where the buffaloes of the Carabao breed were brought by fugitives from French Guiana (1). The buffaloes found an environment similar to their origin, thus being able to survive in an adapted way to the archipelago, which has a predominantly tropical climate, with high environmental temperatures. In general, these animals are conditioned in dual aptitude systems, which seek to maximize the production of meat and milk in a similar way, considering a flexible approach, allowing rural properties, with a focus on livestock, to prioritize both the marketing of milk and meat (2, 3). It is noteworthy that in these systems, animals must seek to adapt to the conditions of interaction, such as the characteristics of their organism and the physical and biotic processes of the environment they surround (4).

The thermal comfort can be defined as a situation in which the thermal balance is zero. The comfort zone for buffaloes is between 15.5 and 21.2°C (5). According to these authors, when buffaloes are subjected to ambient temperature above this parameter, body temperature reacts more strongly, which suggests that this is the upper critical temperature.

Buffalo farming is distributed throughout all Brazilian states and is a viable alternative for milk and meat production (6). In addition, it is not uncommon to find, among us, the buffalo used as a draft animal, as is done in many Asian countries, becoming an animal with multiple functions (7, 8).

Buffalo farming can be considered a relevant activity due to the easy adaptation of those animals to our conditions, docility, rusticity and the high quality of milk and dairy products (9). Even though it is an animal adapted to the local climate, buffaloes are sensitive to the environment and also susceptible to heat stress (10, 11).

Heat stress in buffaloes happens due to some specific characteristics that leave them at a disadvantage when compared to cattle, such as black skin color, black hair, reduced number of sweat glands/skin area, thick layer of the skin epidermis, which makes them vulnerable to solar radiation (12–16). The main consequences of heat stress on the health, production and reproduction of buffaloes are infertility, reduced total milk production, reduced food intake and weight gain (17–19), as well as in the levels of fat, protein and lactose in buffalo milk (20). Buffaloes under heat stress tend to modify their behavioral and physiological responses, and tend to stay most of the time in search of shady areas or seek places where they can wallow in mud to cool off. In addition, they tend to increase water consumption and reduce dry matter consumption, affecting the weight gain of these animals (21, 22).

In this context, monitoring the thermoregulatory response of buffaloes in climatic conditions in the Amazon region becomes essentially necessary to avoid adverse effects of environmental factors on buffalo production. Among the methods of evaluating the surface temperature of buffaloes, the infrared thermographic technique, considered non-invasive, avoids stress to the animal, favoring its well-being (23, 24). Based on this information, the objective of this paper was to evaluate the thermoregulatory responses of buffaloes, depending on the different times of the day, and in systems with and without tree shade, on the island of Marajó, Pará, during the rainy season of the year. In this study, we adopted a hypothesis that even in the wettest season of the year, buffaloes raised on Marajó Island would be in thermal discomfort.

## Materials and methods

### Ethics committee

The experiment was approved by the Ethics Committee, protocol N°. 054/2015 (CEUA) and 23084.013102/2015-01 (UFRA).

### Location

The experiment was carried out on the rural property, located in the municipality of Retiro Grande, Marajó Island, Pará (01°26'S and 48°24'W), during the rainy season of the year (January to April), specifically from February to March. The climate is tropical rainy Am, according to the Köppen classification, with an average annual rainfall of 2,500 mm, an average temperature of 27°C, and a relative humidity of 85% (25).

### Experimental animals

Twenty female buffaloes (*Bubalus bubalis*), non-pregnant and non-lactating, Murrah breed, aged between 2 and 3 years and average weight of 282 ± 29 kg were used. Clinically healthy animals with a body score of three were selected in order to achieve greater uniformity. These animals were kept on pasture and divided into two groups: WS group (with shade) and NS group (without shade). The WS group (*n* = 10) remained in a system with access to the shade of red Jambeiro trees (*Syzygium malaccense*). The NS group (*n* = 10), without access to shade. Both groups had access to water and mineral salt *ad libitum*. The sample used in this study of 10 buffaloes per group was the same used in the Santos et al. (26) and Athaide et al. (27). In this study, only clinically healthy buffaloes were selected. At 6:00, 10:00, 14:00, 18:00, and 22:00 h, for 7 days, animals were taken to the squeeze crush, where they were quickly recorded data from physiological variables that indicate thermal stress: rectal temperature (RT), respiratory rate (RR) and body surface temperature (BST). After 7 days of physiological data collection, buffaloes were changed systems to eliminate animal individual effect. Then, the group that was in the WS system was transported to the NS system and vice versa. After that, animals remained in physiological adaptation again for 2 days in the new system, and then, physiological variables were collected again, for another 7 days.

### Rectal temperature (RT) and respiratory rate (RR)

Rectal temperature (RT, °C) and respiratory rate (RR, mov/min) were measured at 6:00, 10:00, 14:00, 18:00, and 22:00 h, for 7 days. To obtain the RT, a veterinary clinical thermometer (Model-5198.10, Incoterm^®^, São Paulo, Brazil) was used, with a scale up to 44°C, 5 cm was inserted into the animals' rectum for 1 min to measure the RT. The RR was obtained by inspection and direct counting of thoracoabdominal movements, for 1 min, with the aid of a digital stopwatch. These assessments were performed by a single observer.

### Body surface temperature (BST)

BST was obtained with the aid of an infrared thermometer (Model TD-965 - Instrutemp^®^, São Paulo, Brazil) activated at a maximum distance of 1 m from the measurement points on the animal (14), which were: forehead, left side of the thorax and left flank, and the average of those values was obtained at 6:00, 10:00, 14:00, 18:00, and 22:00 h, for 7 days. These measurements were performed by a previously trained veterinarian. After obtaining the temperatures of each region evaluated, the general average of all these regions together by group was calculated.

### Meteorological data

Agrometeorological data were recorded with the aid of a HOBO^®^ data logger, model U30 Station (Onset, U.S.A), installed at the experimental site and the climatic variables measured were air temperature (AT, °C) and relative humidity (RH, 0%). To measure the wind speed (WSP, m/s) a portable digital thermo-anemometer, model TAD – 800 (Instrutherm^®^, São Paulo, Brazil) was used. Solar radiation (RS) was obtained from the INMET meteorological station.

### Temperature and humidity index (THI)

The readings of the environmental variables were performed throughout the day, at the same times of the measurement of the physiological variables. From the values of environmental variables, the Temperature and Humidity Index (THI), proposed by Thom (28), was calculated using the formula:


$$\begin{array}{l} THI=tdb+0,36xtdo+\;41,5 \end{array}$$

Where: tdb = Dry bulb temperature (°C), tpo = Dew point temperature (°C).

### Statistical analysis

The experimental design was a completely randomized factorial crossover, in which all animals were evaluated in two treatments (factor 1 - sun and shade) and at five times (factor 2 −6:00, 10:00, 12:00, 14:00, 18:00, and 22:00 h), in order to eliminate individual influence. The experimental period occurred in two stages, each lasting 5 days, with an interval of 2 days between each stage, for the physiological recovery of the animals, then they changed treatment evaluated at the same five times. Data for physiological (RT, BST, and RR) and climatic variables (THI, WSP, and SR) are expressed as mean and standard deviation.

The physiological statistical model is given by: *yijkt* = μ + *ak* + *bt* + *ci(kt)* + *(ab)kt* + *eijkt*, where *yijkt* corresponds to the response of the i-th animal that received the t-th treatment at the k-th time of the j-th day, μ is the overall mean, ak is the fixed effect of the k-th time of day, bt is the fixed effect of the t-th treatment, ci(kt) is the random effect of the i-th animal on the k- th time of the t-th treatment, (ab)kt effect of the interaction between time and treatment and *eijkt* is the residual error including the random effect; the climate model is given by: *yjk* = μ + *ak* + *ejk*, where *yjk* response obtained at the k-th time of the j-th day, μ is the general average, ak fixed effect of the k-th time and *ejk* is the error residual including the random effect. Statistical analyzes were performed using the SAS v.9.2 program (SAS Institute, Cary, NC, USA, 2010), with normality analysis using the Shapiro-Wilk test and the PROC GLM procedure for the analysis of variance models, having the means of the variables compared by Tukey's HSD test, with 5% probability.

## Results and discussion

There were changes in the meteorological variables between the different times of the day (Table 1), in this context, it was observed that throughout the day the animals are subject to a great intensity of direct solar radiation and short-wave infrared radiation from the ground that give rise to radiant thermal loads above the nuclear temperature itself, which favors the acquisition of heat, which can cause thermal stress (29), when these temperatures are above 25°C (comfort zone ideal temperature) (30), which occurs due to deviations in ambient temperature above the critical upper temperature, causing thermal stress (31, 32). Therefore, heat stress is defined as the sum of environmental and animal factors, that is, the influence of environmental factors associated with inefficient thermoregulation of animals can result in heat stress (33).

**Table 1:** Time and values of the variables wind speed (WSP) and solar radiation (SR) of the climatic variables observed at 6:00, 9:00, 12:00, 15:00, 18:00, and 21:00 h during the experimental period.

**Time**	**Wind speed (m/s)**	**Solar radiation (KJ m^2^)**
6:00 h	0.47c	0.76c
10:00 h	3.82ab	101.02b
14:00 h	4.57a	168.57a
18:00 h	2.60b	4.31c
22:00 h	0.72c	0.60c

Different letters indicate statistical differences (p < 0.05).

There was a highly significant and positive correlation between all environmental and physiological variables (*p* < 0.05) (Table 2). These relationships demonstrate that the environment is considered challenging for the animals, promoting heat stress, because the higher the RR, TR and BST indices, the higher the rate of VV, RS, TA, and RH.

**Table 2:** Correlation between environmental and physiological variables studied for buffaloes.

	**VV**	**RS**	**TA**	**RH**	**THI**
RR RR	0.48267 <0.0001	0.67384 <0.0001	0.64725 <0.0001	0.62110 <0.0001	−0.63318 <0.0001
TR TR	0.46997 <0.0001	0.49042 <0.0001	0.62158 <0.0001	0.59973 <0.0001	−0.64062 <0.0001
BST BST	0.64974 <0.0001	0.77583 <0.0001	0.87891 <0.0001	0.88701 <0.0001	−0.80187 <0.0001

RR, respiratory rate; TR, rectal temperature; BST, body surface temperature; VV, wind speed; RS, solar radiation; TA, air temperature; RH, relative humidity; THI, temperature and humidity index.

**Figure 1:**
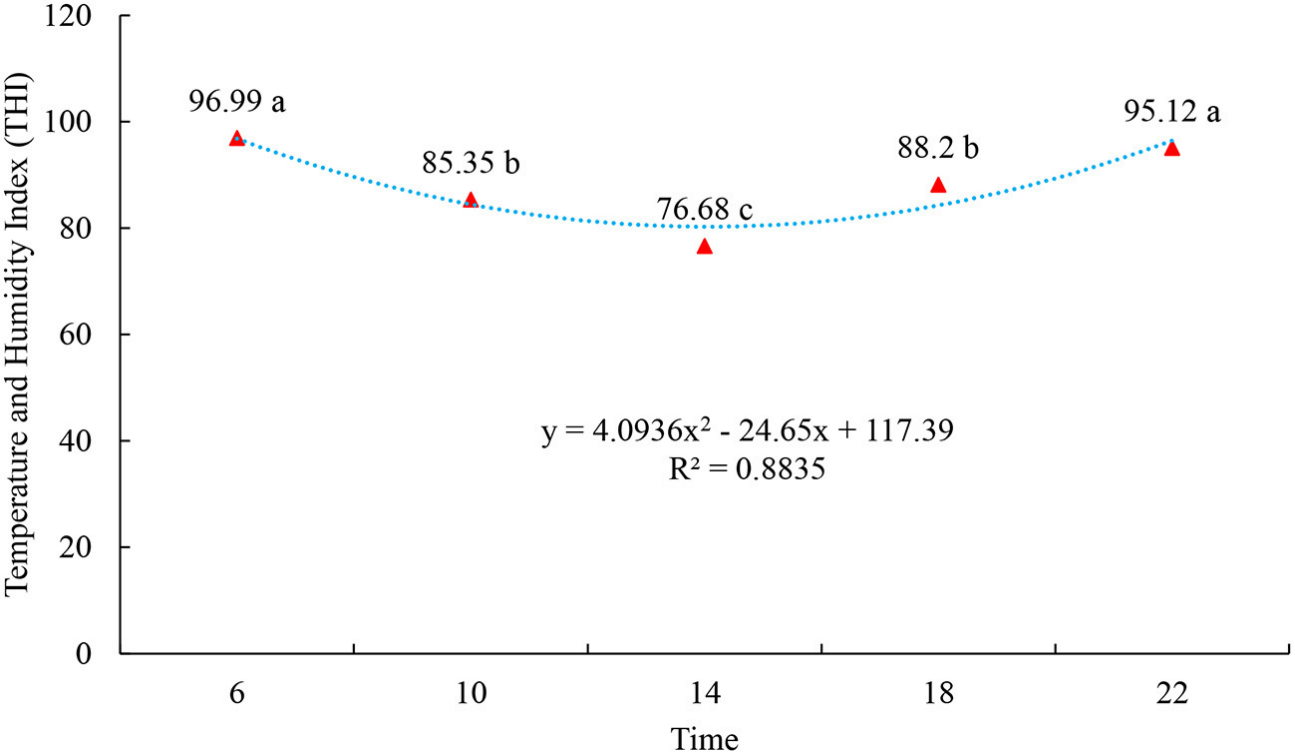
Temperature and Humidity Index (THI) of the climatic variables observed at 6:00, 9:00, 12:00, 15:00, 18:00, and 22:00 h during the experimental period. Different letters indicate statistical differences (*p* < 0.05).

The results indicate that, in the rainy season on the island of Marajó, at all times of data collection, the environment was favorable to cause thermal stress in the animals, as the THI values were high, with the highest THI values occurring at 6 a.m. and 10 a.m.: 00 p.m.: 00 h (*p* < 0.05), probably due to the increase in RH at these times (Figure 1). This can be explained by the fact that high THI and RH can provide greater heat retention, as buffaloes cannot thermoregulate, thus altering respiratory and heart rates, rectal temperature and surface temperature (34). ITU indices tend to vary throughout the year (35), the effects of air temperature are closely linked and dependent on the level of relative humidity, which can affect feeding, ingestion, heat dissipation, creating stressful conditions (36). The THI represents this association well. THI values up to 70 indicate a non-stressful environment, between 71 and 78, critical, between 79 and 83, dangerous and above 83, an emergency condition (37). A study also carried out on the island of Marajó, but in the less rainy period, pointed out that even in challenging conditions under heat stress, buffaloes were able to thermoregulate within the intensity with which there was a reduction in the indices of environmental variables (27). It is noteworthy that thermal stress is one of the environmental problems that makes livestock one of the most challenging activities in different parts of the world, as it directly affects animal production (38).

The WSP variable was higher (*p* < 0.05) at 14:00 h (4.57 m/s), which certainly contributed to facilitate the animals' body heat dissipation by convection and evaporation, at the most stressful times of the day (39). Convective heat loss takes place when a stream of liquid or gaseous fluid, which absorbs thermal energy at a given location, travels to another location, where it mixes with cooler portions of that fluid and transfers thermal energy to them (40).

WSP is of great importance in convective exchanges, with direct involvement in the alteration of physiological variables. Air movement is considered an essential factor for improving environmental conditions, because it influences the loss of heat from the body surface through the evaporation of moisture from the animal's skin (41). For Marai and Haeeb (42), the ideal climatic conditions for growth and reproduction of buffaloes are when WSP is between 5 and 8 km/h.

SR has a direct effect on animals that live in a traditional farming system, without access to shade. It is the factor that contributes to the elevation of AT. We can observe that there was an effect depending on the evaluation time (*p* < 0.05), where at 14 h there was a radiation peak (168.57 KJ m2). During the day, there are less favorable moments for the thermal comfort of the animals, especially when the temperature is above 25°C (30), which can cause thermal stress, which triggers behavioral changes, such as an increase in surface temperature and lower temperature. dry matter intake, and this process is mediated by the radiation balance, that is, by the accounting between the reception and the return of radiation, which is very variable throughout the day. the day and year, which promotes daily and annual changes in TA (43). In addition, the increase in ambient temperature, that is, values above 25°C (30), associated with high humidity can directly influence the growth, reproduction and production of buffaloes, in addition to directly affecting animal welfare (42, 44–46).

Climatic variables can affect metabolism, and consequently, surface temperature and rectal temperature in buffaloes (47), as well as in Nelore (48) and dairy cows (49, 50).

Regarding the physiological variables (Table 3), it is observed that, even in the morning shift, with milder temperatures, the RT values are above the normal range for buffaloes, from 37.4 to 37.9°C, according to Shafie (51). At the times of 10:00, 14:00, and 18:00 h the animals presented higher rectal temperature in buffaloes that were conditioned in the NS group. Body temperature is the result of the difference between the thermal energy produced plus that received by the animal organism and the thermal energy dissipated from these to the environment (52). RT is associated with thermal heat loads in buffaloes (53–58). Higher rectal temperature was observed (39.01°C) at 15:00 in the exposure of female buffaloes to direct sunlight in a hot and humid climate, on Ilha do Marajó, Pará (37).

**Table 3:** Values of average rectal temperature (°C) ± standard deviation off emale buffaloes raised in the sun (NS group) e à sombra (grupo WS), at different times of the day, in the rainy season, on Ilha do Marajó, Pará.

**Time**	**NS**	**WS**
6:00 h	38.06 ± 0.50 Ac	38.20 ± 0.20 Ab
10:00 h	38.64 ± 0.27 Ab	38.36± 0.23 Bba
14:00 h	39.38 ± 0.43 Aa	38.52± 0.19 Ba
18:00 h	38.76 ± 0.22 Ab	38.49 ± 0.23 Ba
22:00 h	38.40 ± 0.13 Acb	38.42 ± 0.19 Aba

Average AB followed by distinct capital letters, in the same line, are different (P < 0.05). Average ab followed by distinct lower case letters, in the same column, are different (P < 0.05).

Body temperature is determined by the difference between the thermal energy produced plus that received by the animal organism and the thermal energy dissipated from these to the environment. At 10:00, 14:00, and 18:00 h, there was a difference in TR between treatments (*p* < 0.05), where the animals of the NS group had the highest values. The lowest temperatures, therefore, were found in animals that had shade available during different times of day. These data show the importance of the availability of trees, in silvopastoral systems, in order to provide shade and improve the thermal comfort of animals in hours of higher solar radiation.

In the NS group, the highest RT value was observed at 2:00 pm (39.38 ± 0.43°C), when SR was highest (168.57 KJ m2). The increase in rectal temperature in a hot environment indicates that the heat release mechanisms have become insufficient to maintain homeothermy (59).

RR is an important thermoregulatory mechanism in buffaloes. Table 4 shows the RR averages of the female buffaloes, at different times of the day, in both treatments. There was a difference in RR between treatments at 10:00, 14:00, and 18:00 h (*p* < 0.05), where the highest values were found in the NS group (33.71 ± 7.58 and 50.40 ± 18.41 and 22.00 ± 1.55 movements/min., respectively), demonstrating the importance of providing shade to the animals during the hottest hours of the day. Daltro (60) obtained RR values from 40 to 60, 60 to 80, and 80 to 120 mov/min and characterizes, respectively a low, medium and high stress for ruminants, and above 200 mov/min the stress is classified as severe.

**Table 4:** Values of average respiratory rate (mov./min.) ± standard deviation of standard female buffaloes raised in the sun (NS group) and in the shade (WS group), at different times of the day, in the rainy season, on Ilha do Marajó, Pará.

**Time**	**NS**	**WS**
6:00 h	19.90 ± 2.55 Ac	18.68 ± 1.25 Ab
10:00 h	33.71 ± 7.58 Ab	23.78 ± 2.68 Ba
14:00 h	50.40 ± 18.41 Aa	24.20 ± 3.70 Ba
18:00 h	22.00 ± 1.55 Ac	19.61 ± 1.95 Bb
22:00 h	18.64 ± 1.93 Ac	18.41 ± 1.88 Ab

Average AB followed by distinct capital letters, in the same line, are different (P < 0.05). Average ab followed by distinct lower case letters, in the same column, are different (P < 0.05).

The highest RR values were verified for the treatment in full sun and at 14:00 h, which indicates a thermal stress condition for the animals at that time. The increase in RR is important for endogenous heat dissipation, however, it demands energy expenditure, which implies more physiological damage for the maintenance of the animal (61). Thus, under conditions of heat stress, buffaloes tend to perform peripheral vasodilation, increasing blood flow to the skin surface, causing more profuse sweating and, consequently, an increase in respiratory rate (62–65).

In the evaluation of the average data, in the WS group at all times and in the NS group at 6:00, 18:00, and 22:00 h, the observed values are within the normal variation range for buffaloes, from 18 to 30 mov./min. (51). On the other hand, the values observed in the NS group, at 10:00 and 14:00 h, surpassed those indicated for situations of thermoneutrality of the buffalo species, which was already expected, since the combination of climatic elements caused a greater degree of discomfort. to the animals, raising the RR in order to maintain body temperature at normal levels. The mechanisms that provide respiratory and skin cooling are associated with heat dissipation by the body, emitting more moisture to the environment (66, 67). The increase in temperature was recorded in different breeds of cattle, such as Angus, Nelore and Sahiwal, subjected to high temperatures (68).

Regarding the BST evaluated, the averages are shown in Table 5. At the times of 10:00 and 14:00 h, BST was higher in the SS group (*p* < 0.05), from 34.55 ± 1.03 and 35.35 ± 1.51°C, respectively, and both groups had higher TSC values at the same times. Therefore, RS tends to directly influence the orbital region (*regio orbatalis*), dorsal region (*regio dorsi*), scrotal region (*regio scrotalis*), udder region (*regio uberis*) and mammary gland region, as evidenced by infrared thermography in the main areas of the eye sockets and in the muscles of the spine, scrotum or mammary gland (69, 70), or in an environment with or without tree shade (11, 28). The regions selected for evaluation in this study were chosen because they have a small amount of fur and, therefore, present greater precision in the surface temperature of the animals studied. Because areas of the body with long hair can cause thermal insulation of the body, and present altered results (71).

**Table 5:** Values of average (°C) body surface temperature (BST) ± standard deviation of standard female buffaloes raised in the sun (NS group) and in the shade (WS group), at different times of the day, in the rainy season, on Ilha do Marajó, Pará.

**Time**	**NS**	**WS**
6:00 h	30.57 ± 1.33 Ac	30.22 ± 1.06 Ac
10:00 h	34.55 ± 1.03 Aa	33.66 ± 0.85 Ba
14:00 h	35.35 ± 1.51 Aa	33.75 ± 0.85 Ba
18:00 h	32.42 ± 0.84 Ab	31.84 ± 1.10 Ab
22:00 h	30.52 ± 1.27 Ac	30.94 ± 0.86 Acb

Average AB followed by distinct capital letters, in the same line, are different (P < 0.05). Average ab followed by distinct lower case letters, in the same column, are different (P < 0.05).

These results demonstrate that exposure to full sun, due to the incidence of solar radiation, raised the body surface temperature by 1.6°C, compared to the treatment with availability of shade, at 14:00 h, as buffaloes prefer to feed in shaded areas (10). With similar results, Santos et al. (26), in the Amazon, observed that buffalo heifers looked for shaded areas, especially in the hottest hours of the day, to ruminate, both standing and lying down, in search of the most suitable place for their well-being. The radiant energy load incident on the animal, in tropical regions, can be greater than three times the total endogenous heat produced by the animal itself. Thus, the absorption of solar radiation by the animal and the ambient temperature can increase the production of metabolic heat, resulting in thermal discomfort (11). Research carried out evaluating the thermoregulatory mechanism of buffaloes describes shade as a relevant component to enhance heat dissipation in different environments (26, 28, 72, 73). In this sense, heat stress can promote a reduction in grazing activity (74), which can cause weight loss in cattle (75).

In this study, a limitation was the non-use of infrared thermography, which is a tool to assess specific regions with vasomotor alterations mediated by the autonomic nervous system. However, we ended up using the laser thermograph to assess the surface temperature. In this sense, the published scientific evidence allows its use to be recommended (69), as it is a tool that has greater sensitivity, specificity and analysis capacity than the infrared thermometer, which is why it represents a broader field of application that can continue to be explored in the future.

## Conclusions

Even in the rainy season of the year, the climatic conditions of the island of Marajó are unfavorable to the well-being of the buffaloes, as they presented altered environmental parameters, differing between the groups, as well as physiological changes in rectal and surface temperature, and respiratory rate, mainly me buffaloes conditional to the sun. In addition, these changes were more evident, especially at 2 p.m., when solar radiation is more intense, thus, the use of trees in the pasture is indicated, in order to provide shade and greater thermal comfort to the animals and, consequently, improve their productivity.

## Data availability statement

The raw data supporting the conclusions of this article will be made available by the authors, without undue reservation.

## Ethics statement

The animal study was reviewed and approved by the Ethics Committee, protocol no. 054/2015 (CEUA) and 23084.013102/2015-01 (UFRA).

## Author contributions

Experiment design: JS and JL. Experiment execution: JS, MP, JA, and RN. Data curation: AB, JS, and WS. Formal analysis: WS and AB. Original writing: JS, WS, AB, and JL. All authors edited and approved the final manuscript.

## Funding

This study was funded in part by the Federal University of Pará and Coordenação de Aperfeiçoamento de Pessoal de Nível Superior (CAPES) Brasil (Finance Code 001). Also, received financial support for the publication fee from the Pró-Reitoria de Pesquisa e Pós-Graduação (PROPESP/UFPA).

## Conflict of interest

The authors declare that the research was conducted in the absence of any commercial or financial relationships that could be construed as a potential conflict of interest.

## Publisher's note

All claims expressed in this article are solely those of the authors and do not necessarily represent those of their affiliated organizations, or those of the publisher, the editors and the reviewers. Any product that may be evaluated in this article, or claim that may be made by its manufacturer, is not guaranteed or endorsed by the publisher.
